# Interspecies Gene Transfer as a Method for Understanding the Genetic Basis for Evolutionary Change: Progress, Pitfalls, and Prospects

**DOI:** 10.3389/fpls.2015.01135

**Published:** 2015-12-22

**Authors:** Lachezar A. Nikolov, Miltos Tsiantis

**Affiliations:** Department of Comparative Development and Genetics, Max Planck Institute for Plant Breeding ResearchCologne, Germany

**Keywords:** *Cardamine hirsuta*, evolution of morphology, leaf development, regulatory evolution

## Abstract

The recent revolution in high throughput sequencing and associated applications provides excellent opportunities to catalog variation in DNA sequences and gene expression between species. However, understanding the astonishing diversity of the Tree of Life requires understanding the phenotypic consequences of such variation and identification of those rare genetic changes that are causal to diversity. One way to study the genetic basis for trait diversity is to apply a transgenic approach and introduce genes of interest from a donor into a recipient species. Such interspecies gene transfer (IGT) is based on the premise that if a gene is causal to the morphological divergence of the two species, the transfer will endow the recipient with properties of the donor. Extensions of this approach further allow identifying novel loci for the diversification of form and investigating *cis*- and *trans*-contributions to morphological evolution. Here we review recent examples from both plant and animal systems that have employed IGT to provide insight into the genetic basis of evolutionary change. We outline the practice of IGT, its methodological strengths and weaknesses, and consider guidelines for its application, emphasizing the importance of phylogenetic distance, character polarity, and life history. We also discuss future perspectives for exploiting IGT in the context of expanding genomic resources in emerging experimental systems and advances in genome editing.

As species diverge, so do their genomes and morphologies. Regulatory evolution and consequent modifications of transcriptional networks of a broadly conserved repertoire of developmental genes are believed to be at the heart of evolutionary change in morphology (Carroll, 2008; Peter and Davidson, 2011). Regulatory changes involve modification of *cis*-regulatory elements and the *trans*-environment, and understanding these processes is critical for understanding how traits diversify. However, to pinpoint the precise genetic changes that underlie morphological diversity at different evolutionary scales remains a fundamental challenge. One way forward is to follow a transgenic approach, and transfer a gene suspected to contribute to the divergence between two species with contrasting morphologies. Such interspecies gene transfer (IGT) is based on the premise that if a gene is causal to divergence, the transfer will endow the recipient with properties of the donor (Figure 1A).

**Figure 1:**
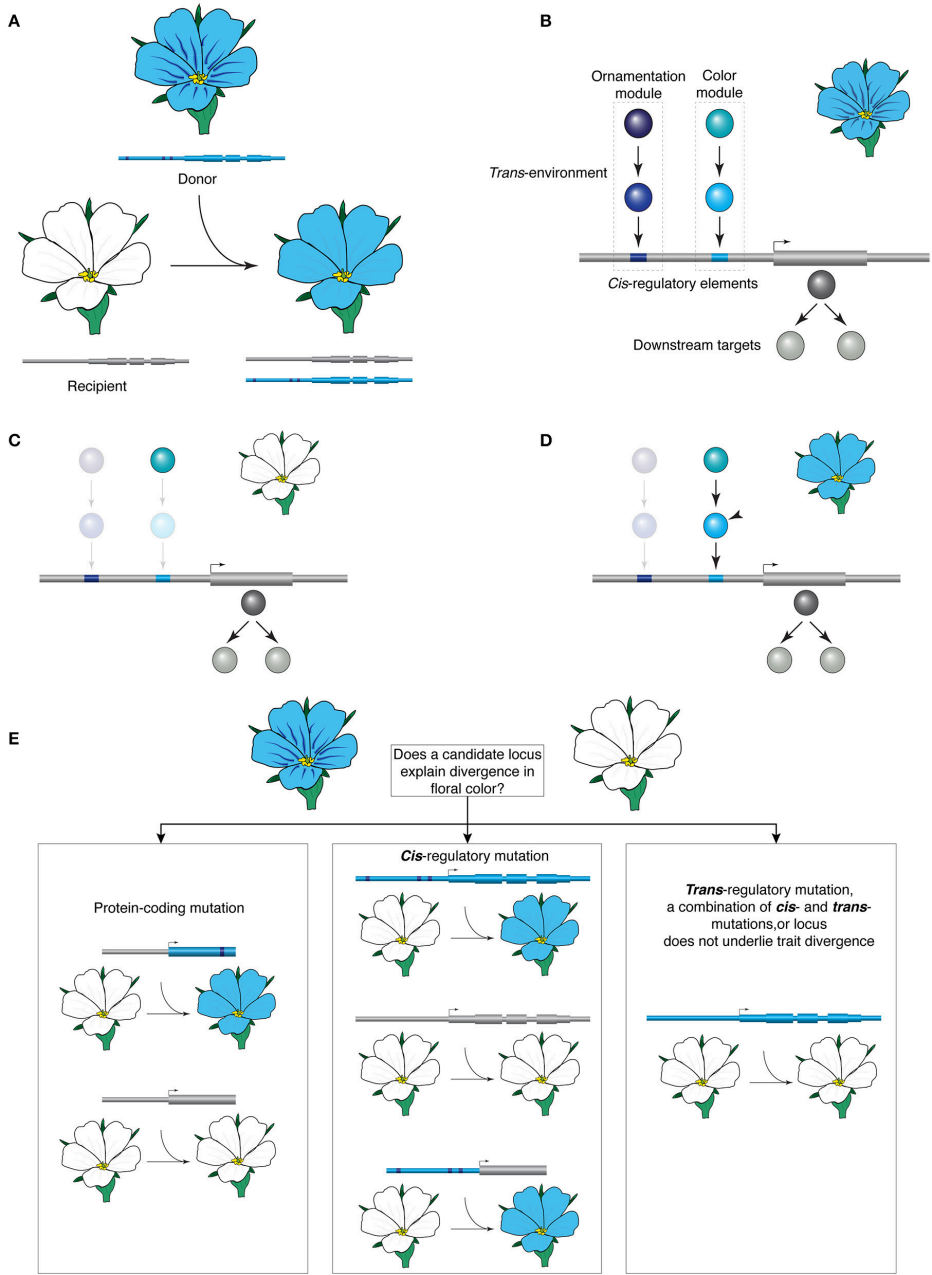
**The premise of interspecies gene transfer. (A)** Transferring a locus from the blue-flowered donor species is able to transform the color of the white flowers of the recipient; note that the transfer does not recapitulate the ornamentation (the nectar guides) of the donor in the recipient. **(B–D)** Hypothetical network controlling color and ornamentation expression. **(B)** Light and dark blue transcription factors (TFs) interact directly with the *cis*-regulatory elements of a key blue pigment synthesis gene, and control the color and the ornamentation of the blue flower, respectively. These TFs are in turn regulated by upstream regulators. **(C)** In the white-flowered species, pigment synthesis is abolished via inactivation mutations in TFs but the blue pigment synthesis genes (gray) and the upstream regulator for blue color (light blue) remain intact. **(D)** Introducing the light blue TF (arrowhead) restores blue pigment synthesis in a white-flowered recipient but does not transfer the ornamentation pattern. **(E)** Possible outcomes of an IGT experiment designed to test the contribution of a candidate locus to the divergence in color. In protein divergence, expression of the coding sequence of the donor under the recipient's promoter will result in change of phenotype. If *cis*-regulatory evolution underlies phenotype divergence, the coding sequence of the recipient expressed under the donor's promoter and the entire locus of the donor may be sufficient for phenotypic change. *Trans*-regulatory mutation, a combination of *cis*- and *trans*-mutations, or lack of involvement of the locus are possible when transferring the entire locus of the donor does not reconstitute the phenotype in the recipient.

## The premise

IGT is a functional test for sufficiency to study the underlying genetic basis of trait divergence and can be used between species that do not hybridize. As such it complements the classical approach based on genetic crosses. Examining the contribution of the transgene on the trait under study can indirectly provide information about its underlying genetic architecture (Figures 1B–D). The transfer of the entire interrogated locus, including its non-coding regulatory elements, allows studying evolutionary events concerning both coding and regulatory sequences and these have distinct outcomes in the context of IGT (Figure 1E). Protein divergence underlies the trait divergence in two species when the coding sequence of one species is able to elicit a phenotypic change in the other species, whereas the endogenous copy under the same promoter does not (see Kramer, 2015 for details). To further characterize biochemical divergence, which can manifest as a metabolic difference or as differences in the expression of downstream genes, the amino acid differences between the two proteins can be interrogated, for example in *in vitro* assays (e.g., Hoekstra et al., 2006). When two species diverged morphologically but the protein function did not change during evolution, expression difference underlying the divergence is suspected. In this case, coding sequences from both species under the same promoter may be able to elicit phenotypic change in the recipient but if a *cis*-regulatory change is causal, only the entire locus from the donor will be similarly potent. Alternatively, if transfer of the entire locus from the donor has no detectable effect on the recipient's morphology, *trans*-regulatory change, a combination of *cis*- and *trans*-changes, and downstream gene divergence are plausible explanations. In all cases, the experiment should be interpreted in the context of other critical data, such as loss-of-function phenotypes, expression analyses, and the phylogenetic distribution of character states.

Transfer of a heterologous locus into the recipient genetic background will result in a phenotypic change when three criteria are satisfied. First, the encoded protein can perform biochemically; second, it is expressed in the correct (or at least developmentally meaningful) time and place; and third, enough of the gene regulatory network for the trait is intact in the recipient. On the other hand, insufficient dominance resulting for example from absence of synergistic activities, and substantial divergence owing to co-evolution between *cis*-elements and *trans*-factors will render the heterologous locus non-functional. Thus, in its current use, IGT is a one-by-one locus approach that is not well suited for assessing the degree of functional and causal interdependence between endogenous genes in the donor. One of the first transgenic studies to understand the genetic basis of morphological evolution in animals examined the wing pigmentation of fruit flies in the *melanogaster* group (Gompel et al., 2005). To understand the origin of a novel wing spot, *yellow* 5′ regulatory sequence of *Drosophila biarmipes*, which features a spot, was fused to a reporter and introduced into the spot-free *D. melanogaster*. The reporter displayed expression pattern similar but not identical to the one observed in the donor *D. biarmipes*, which suggests that divergence at the *yellow* regulatory region contributed to the novel wing pigmentation pattern. It also revealed additional *trans*-factors that confer the precise spatio-temporal expression of the spot (Gompel et al., 2005). Furthermore, introducing a partial *yellow* locus of *D. biarmipes* was not sufficient to generate a spot in *D. melanogaster*, indicating that additional loci are involved.

## Other notable applications

**Figure 2:**
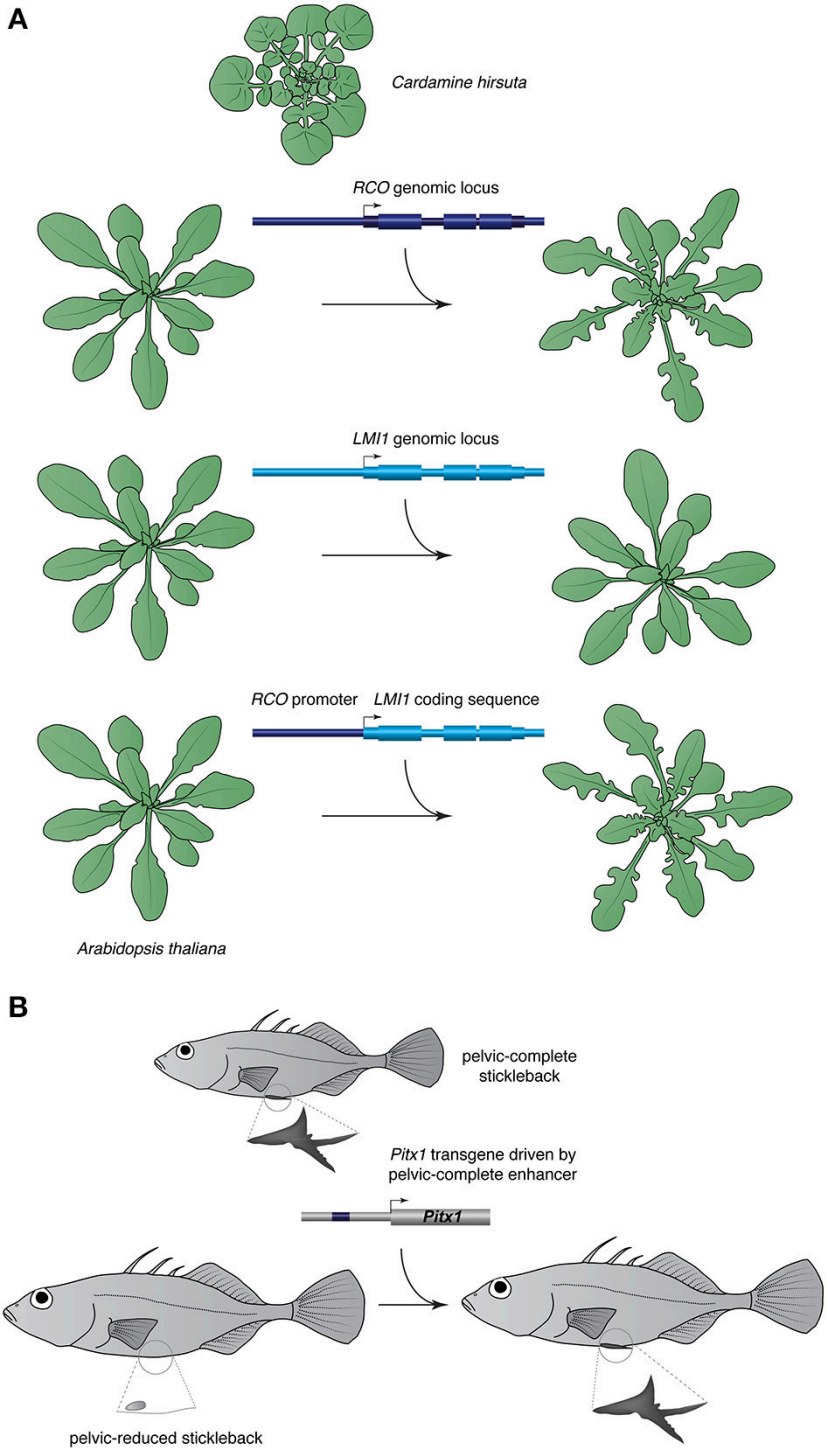
**Examples of IGT. (A)** *RCO* affects leaf morphology and a copy of the gene from the compound leaved mustard *Cardamine hirsuta* is able to transform the simple leaves of *Arabidopsis thaliana* into complex lobed leaves. A genomic locus of the paralog of *RCO, LMI1*, transformed in *A. thaliana* is not able to modify leaf morphology but plants expressing *LMI1* driven by the *RCO* promoter have lobed leaves, suggesting regulatory divergence of the two paralogs. **(B)** *Pitx1* enhancer affects pelvic morphology in sticklebacks; transfer of the enhancer and coding sequence of pelvic-complete stickleback into a pelvic-reduced stickleback results in pelvic expression and development.

IGT is a powerful test for the contribution of candidate loci known to affect a given trait in other species. Extensive use of the method in an evolutionary context has been made in studies of the evolution of angiosperm leaf shape (Figure 2A; Hay and Tsiantis, 2006; Barkoulas et al., 2008; Vlad et al., 2014; Rast-Somssich et al., 2015). These experiments showed that two apparently independent developmental modules contribute to leaf complexity in the family Brassicaceae. One involves class I *KNOTTED1*-like homeobox (*KNOX*) genes where *cis*-regulatory changes underlie the divergence between the simple-leaved *A. thaliana* and its compound-leaved relative *Cardamine hirsuta* (Hay and Tsiantis, 2006; Barkoulas et al., 2008; Rast-Somssich et al., 2015). Transferring *KNOX* gene paralogs from *C. hirsuta* into *A. thaliana* provides evidence for an inverse relationship between pleiotropy of a gene and its potential to evolve variants able to alter morphology in an IGT experiment (Rast-Somssich et al., 2015). The other involves the *REDUCED COMPLEXITY* (*RCO*) homeobox gene, which is a member of a tandem three-gene cluster in many mustard species (Vlad et al., 2014). Having lost *RCO* from its genome, which likely contributed to leaf shape simplification, *A. thaliana* retains only one member of this cluster, *LATE MERISTEM IDENTITY 1* (*LMI1*). In *C. hirsuta, RCO* and *LMI1* are expressed in near complementary domains, the former at the base of developing leaflets, and the latter along leaflet margins and in the stipules, respectively. Importantly, when expressed from the *RCO* promoter both *RCO* and *LMI1* coding sequences can complement the *C. hirsuta rco* mutant, which exhibit simplified leaves, supporting the idea that regulatory rather than coding divergence underlies the functional differences of these two paralogs (Vlad et al., 2014). Using transformation to move the entire genomic *RCO* locus from *C. hirsuta* into *A. thaliana*, which is in principle a functional equivalent of the *C. hirsuta rco* mutant, produces deep lobes in the otherwise nearly smooth leaf margin of *A. thaliana*. Similar results were obtained with the *Capsella* homolog of *RCO* (Sicard et al., 2014). Thus, an introduction of a single gene is capable to reverse-engineer a character lost in *A. thaliana*. The *C. hirsuta RCO* locus is able to modify the simple leaf of *A. thaliana* likely because it represents the derived state due to *RCO* loss but retains the ancestral regulatory landscape that promotes leaf complexity through *RCO* activation. This renders *RCO* a major effect locus that may account for much of the variation in leaf shape in Brassicaceae (Sicard et al., 2014; Vlad et al., 2014).

That reintroduction of a single gene can restore a morphological state was also demonstrated in threespine stickleback fish, where reductions or loss of the pelvic girdle and spines, which feature prominently in marine populations, have been lost several times independently after freshwater transition (Figure 2B; Chan et al., 2010). Linkage mapping has identified a region containing the *Pituitary homeobox 1* (*Pitx1*) gene to account for much of the variance in pelvic size, and although Pitx1 protein sequence in pelvic-reduced sticklebacks is identical to their marine ancestors', its expression is abolished in the pelvic region, suggesting a causal regulatory mutation (Shapiro et al., 2004). The mutation was mapped to a deletion in the upstream noncoding region of *Pitx1* in pelvic-reduced sticklebacks that contains a tissue-specific enhancer (Chan et al., 2010). Introducing the *Pitx1* enhancer and coding sequence into fertilized eggs of pelvic-reduced fish resulted in enlarged pelvic girdle and external pelvic spine in transgenic fish, demonstrating the functional significance of *Pitx1* in pelvic development.

The *Pitx1* and *RCO* examples highlight the advantage of recipients with loss-of-function phenotypes in transgenic rescue experiments. Derived gain-of-function phenotypes can also be transferred to provide evidence for sufficiency. For example the trait of four abdominal bristles from *Drosophila quadrilineata* can be transferred to the two-bristled *D. melanogaster* via the *scute* enhancer from *D. quadrilineata* but not via transferring homologous enhancers from species with only two abdominal bristles (Marcellini and Simpson, 2006). In another example, transferring the promoter and coding sequence of the plasma membrane ATPase *HMA4* from *Arabidopsis halleri*, which exhibits heavy metal hyperaccumulation to the non-accumulator *A. thaliana* resulted in increased *HMA4* transcript levels (Hanikenne et al., 2008). The transgenic *A. thaliana* plants also showed zinc distribution in the root comparable to *A. halleri* suggestive of zinc partitioning and tolerance, but toxic shoot zinc hypersensitivity characteristic of wild type *A. thaliana*. This finding indicates that additional genes are necessary to reconstitute all facets of the hyperaccumulator syndrome in plants.

In a study designed to investigate a possible contribution of the *LEAFY (LFY)* transcription factor to the divergence of plant architecture in Brassicaceae, the entire *LFY* locus from the rosette flowering crucifers *Ionopsidium acaule* (*IacLFY*), *Idahoa scapigera* (*IscLFY1*), and *Leavenworthia crassa* (*LcrLFY*) was independently introduced into *A. thaliana lfy-6* mutant background, which shows defects in floral meristem identity (Yoon and Baum, 2004). The *IacLFY* locus was able to rescue the *lfy* phenotype as expected for regulatory and protein conservation and thus cannot explain rosette flowering in *I. acaule*. In contrast, *IscLFY1* rescued some aspects of the *lfy* floral phenotype in *A. thaliana*, but generated developmental defects, such as bracteate flowers (bracts normally abort in Brassicaceae), shortened internodes, and occasionally aerial rosettes resembling the phenotype of the donor, suggesting that *IscLFY1* may contribute to rosette flowering (Yoon and Baum, 2004). Similarly, *LcrLFY* partially rescued the floral *lfy* phenotype but some transgenic lines produced terminal flowers as in wild *L. crassa* plants. These observations imply different mechanisms for rosette flowering in the studied species, but the complex transgenic phenotypes make interpretation difficult, likely because *LFY* affects many downstream processes beyond plant architecture (Winter et al., 2011). Pleiotropic effects may hinder donor phenotype reconstitution using developmental master regulators even when a complex phenotype is reduced to well-defined principle components. Despite rigorous phenotypic analysis, a study assessing the contribution of the transcription factors *doublesex* and *fruitless*, which coordinate sex-specific functions, to species-specific male courtship dance revealed that although transgenes from four *Drosophila* species were able to rescue *D. melanogaster* courtship behavior, no elements of the ritualized species-specific dance were transferred (Cande et al., 2014).

The test for sufficiency can be extended to a forward screen to find novel genes contributing to morphological divergence between species, as initially proposed under the term transgenomics (Baum, 2002; Correa and Baum, 2015). A proof-of-concept study reported the introduction of ca. 4% of the genome of *Leavenworthia alabamica*, a relative of *A. thaliana* that differs in a number of traits, into *A. thaliana* to screen for changes in morphology consistent with the presence of a transgene (Correa et al., 2012). The technique holds much promise when larger portions of the genome are introduced into the donor and more primary transformants are screened. A transgenomic screen of a large insert library from the salt tolerant mustard *Eutrema salsuginea* into *A. thaliana*, which represent two divergent lineages in the mustard family, revealed a stress tolerant candidate locus (Wang et al., 2010). A similar study to identify factors for drought and alkaline tolerance of the resurrection plant *Boea hygrometrica* (Gesneriaceae), an asterid, as a donor and the rosid *A. thaliana* as a recipient revealed a retro-element fragment conferring improved photochemical efficiency and membrane integrity under osmotic and alkaline stress (Zhao et al., 2014). Although, the precise mechanisms by which these loci confer tolerance are currently unknown, this approach has a potential to identify putative causative variants that can be examined further.

## Practical considerations

IGT is a versatile tool and can be applied in both forward (i.e., transgenomics) and reverse genetics context to obtain functional information for genes identified in forward genetic screens (e.g., EMS mutagenesis screens), as well as candidates from comparative gene expression studies and targeted transcriptome profiles (e.g., from laser capture microdissection- and INTACT-derived cell specific transcriptomes; Nelson et al., 2006; Deal and Henikoff, 2011). In that respect, the technique can be used successfully to study both homologs of known morphologically important genes, as well as non-obvious candidates identified through high-throughput genomic and transcriptomic approaches. IGT is most revealing in combination with data on gene expression and the biochemical conservation of the protein. The native expression of the locus in the donor and its reconstituted expression in the recipient can be characterized by promoter reporters to determine whether the transgene will be expressed in a functionally relevant position (i.e., corresponding to the donor's) and to further infer *cis*-regulatory changes in the promoter or *trans*-changes in factors upstream of the candidate locus in the genetic hierarchy. To test the biochemical potential of the protein to alter form, the coding sequence of the donor species can be expressed under a broadly active promoter (e.g., *CaMV 35S, pRPS5a*, and *Ubi* promoters) in the recipient species. Many eukaryotic genes are alternatively spliced, and to reduce cloning efforts and avoid bias in calling splice variants, constructs containing the entire exon-intron structure between the start and the stop codon can be transferred into the recipient species to allow processing by the endogenous splicing machinery. This approach may also allow identification of control elements, such as intronic enhancers. Since the resulting phenotype may be difficult to interpret due to pleiotropic defects that reflect expression that is too broad in space or time, or has a very high level, expression in a narrower domain known for its strong morphogenetic properties, such as leaf margin (Hagemann and Gleissberg, 1996), vertebrate limb bud, and insect imaginal disk may be more suitable to assess biochemical function. However, implicit assumption of when and where the protein is expressed may not be fully congruent with its native pattern of expression, and may prevent the protein from eliciting function. Transferring the entire locus, including its coding sequence and regulatory elements will reconstitute the spatiotemporal context where the protein operates in the donor if it is functional in the recipient's *trans*-environment.

In plants, the IGT constructs are generally introduced into the donor genome using *Agrobacterium*-mediated transformation. A transformation event often results in introducing multiple copies of the transgene so studying single T-insertion lines in detail is preferable. Since dosage alone can account for much of the observed phenotypic change, it is critical to confirm that such effects are not causal by independently transforming the recipient's endogenous copy as a control and comparing the phenotypic distribution. Another useful control is introducing the transgene into recipient's null mutant background in a complementation test; however, few species outside of the established models permit such experiment. To avoid positional effects and circumvent transgene silencing, multiple independent T-insertions are to be analyzed, and rare phenotypes should be treated with caution.

## Critical appraisal

IGT is particularly valuable for determining the genetic basis for morphological variation between reproductively isolated species when classical genetics methods including QTL analysis are not feasible. Genetic transformation is a prerequisite for IGT. Since the introduced transgene is in hemizygous state and resides in the recipient genome along with the endogenous copy, typically only gain-of-function phenotypes are accessible. As such, IGT is a test for sufficiency, which can determine whether the introduced copy alone has the potential to recreate and is thus likely causative to the donor phenotype. Based on these assumptions, the IGT strategy is particularly powerful if the donor represents the ancestral state of the studied character, and the recipient exhibits a loss-of-function derived state. Reconstructing the ancestral phenotype then also suggests that the rest of the transcriptional network underlying the trait (or a network that is functionally equivalent) is intact in the recipient. Alternatively, a donor with derived character state may elicit phenotypic response in a recipient that lacks the trait (ancestral state) if the introduced locus can function alone (e.g., many metabolic enzymes), or is capable of coopting appropriate downstream targets. The second scenario is most likely if the gene regulatory hierarchy of the trait is not particularly complex and the number of loci involved is not too large. Thus, knowledge of character polarity distribution is useful in the experimental design and interpretation of IGT. As directionality of evolutionary change is often difficult to infer from incompletely sampled phylogenies, extending the common garden experiment in ecology to genetics (i.e., swap of promoters or entire loci between a donor and a recipient in an equivalent of a reciprocal transplant) circumvents the need to understand the phylogenetic distribution of morphological states (Hay and Tsiantis, 2006; Kellogg, 2006a,b; Gordon and Ruvinsky, 2012). Because we are often constrained to a particular focal species as a donor, there is more flexibility in selecting the recipient species—the more advanced experimental model in a given phylogenetic proximity is a reasonable choice. While it is impossible to select a recipient that differs from the donor only by the character under study or to introduce the transgene in an ancestor prior to the acquisition of the character state, related species with similar life history and growth habit can be used to obtain an interpretable phenotypic readout. In plants, the model *A. thaliana* can serve as a reference recipient species in many IGT studies due to practical considerations, such as reliable transformation, rapid life cycle, and lack of prolonged seed dormancy. However, the IGT outcomes are particularly sensitive to the choice of donor and recipient species (Ruvinsky and Ruvkin, 2003; Barriere and Ruvinsky, 2014; Gordon et al., 2015).

A major issue with the IGT approach is the interpretation of the resulting phenotypes. The approach attempts to atomize a trait by interrogating an individual locus and its contribution, which is an advantage for interpreting gene transfers of major phenotypic and minimal pleiotropic effects. Traits with simpler underlying genetic architecture, such as ones determining certain physiological traits are readily amenable to study by IGT (Hanikenne et al., 2008; Wang et al., 2010). These traits appear less sensitive to the phylogenetic distance between the donor and recipient genomes, and phenotypic changes that are more straightforward to quantify. In complex traits evolved by accumulation of many changes, sequence divergence and the co-evolution of *cis*- and *trans*-elements becomes more substantial and the contribution of any individual locus diminishes, which confounds interpretation. Similarity in body plan, life history, and growth habit, and the ability to make clear homology statements improve interpretability. With increase of phylogenetic distance, homology inference for at least some characters becomes more difficult. This is a reason why there are limitations to the conclusions that can be drawn from transfer of genes between distantly related species with widely divergent morphologies, for example when attempting to understand the origin of flowering by transferring orthologs of seed plant floral identity genes from mosses into *A. thaliana*, though such experiments can inform on the biochemical potential of the proteins in questions in divergent lineages (Maizel et al., 2005). A measure of functional conservation of the gene regulatory machinery at a phylogenetic scale might be obtained from reporter gene analyses, either through reciprocal swap of 5′ regulatory sequences, or via expression of heterologous regulatory sequence in a single reference species (Kalay and Wittkopp, 2010). Reporter expression demonstrated limited conservation of regulatory sequence and/or *trans*-factors between *Drosophila melanogaster* enhancers that were moved into *Caenorhabditis elegans* by transformation, and extensive conservation in swaps between *C. elegans* and *C. briggsae*, with several specific instances of functional divergence (Ruvinsky and Ruvkin, 2003). Similar experiments with the regulatory sequences of eight genes from four *Caenorhabditis* nematodes in *C. elegans* revealed overall conservation of expression pattern with broader expression in other cell types, which was interpreted as a sign of functional divergence (Barriere and Ruvinsky, 2014). Similar trend with at least partial conservation of gene expression patterns not reflected in sequence similarity persists at a phylum level for nematodes that diverged more than 400 million years ago (Gordon et al., 2015). Therefore, depending on the complexity of the trait under study the approach can be applicable at various scales and informed judgment that considers lineage specific evolutionary patterns and rates is needed to decide on the most appropriate experimental setup.

## Summary and future prospects

Traditionally, identifying the loci underlying trait divergence is based on crosses between two populations or species with contrasting morphologies and examining the phenotypic distribution of traits in the progeny. The precise proportion of gene activity that accounts for morphological divergence of reproductively isolated species, however, is difficult to conceptualize. IGT provides a platform to address this question transgenically by interrogating candidate loci in their entirety and via their regulatory and protein-coding components. A weakness of the approach is the underlying assumption that a certain aspect of the trait can be reducible to a single gene (see Baum, 2013, for a discussion on the causation between individuated traits and developmental-causal genes, and Orgogozo et al., 2015), which is only an operational approximation that will have limited validity when multigene interactions underlie the diversification of the studied trait. To offset this shortcoming, an extension of the method to introduce several transgenes into the recipient, which collectively may reconstruct a functional module underlying the trait under study, can be applied. Some of the characteristics of the transgenic approach, such as dosage and integration effects, which obstruct interpretability, can be overcome by gene replacement of an endogenous gene with a transgene placed in comparable genomic position via homologous recombination (Puchta, 2002). Not all species are amenable to gene replacement, which makes emerging technologies in genome editing (e.g., programmable nucleases and the CRISPR-Cas system, which allow direct and precise manipulation of gene function) particularly promising to specifically target loci in their endogenous genomic context. The explosion of sequencing information from a wide range of organisms should greatly facilitate the broad application of IGT (Rowan et al., 2011). Sequencing information in combination with improved tools for genome editing will advance the versatility of the platform through introducing judiciously distributed species pairs at key phylogenetic positions (Jenner and Wills, 2007; Abzhanov et al., 2008). The progress in editing technologies also allows for a more straightforward application of other techniques used to assess gene contribution to phenotypic change, such as the reciprocal hemizygocity test, which compares the phenotypes of reciprocal hybrids that are genetically identical throughout the genome except at the test locus (Stern, 2014). In addition to opening new avenues for comparative research and contributing to the shift from studying the pattern of variation to providing a mechanistic insight into the genetic basis of evolutionary change, IGT also offers the conceptual background for the reverse engineering of traits of practical interest through synthetic biology.

## Author contributions

LN and MT wrote and approved the manuscript.

### Conflict of interest statement

The authors declare that the research was conducted in the absence of any commercial or financial relationships that could be construed as a potential conflict of interest.
